# Transactions of the Pathological Society

**Published:** 1842-10-22

**Authors:** 


### MEDICAL EXAMINER.

NEW SERIES.

No. 43.] PHILADELPHIA, OCTOBER 22, 1842. [Vol. I.

### TRANSACTIONS OF THE PATHOLOGICAL SOCIETY.

*Monday Evening, October 17th.*

The President, Dr. Chapman, in the Chair. Dr. Stillé read the report of

### *A Case of Tri-facial Neuralgia, cured by extracting a carious Molar Tooth.*

Elizabeth McMakin, aged about 27 years, has been in good health since October, 1840, when she was ill with continued fever. She is naturally robust, and has never been subject to rheumatism or shooting pains in any part of her body. About a year ago she suffered from toothache in one of the upper molar teeth of the right side; the pain was confined to a single tooth, and ceased upon the extraction of the latter, which was found free from decay.

On the 29th September, 1842, she ate her supper as usual, masticating her food readily with the teeth of both sides. A few hours afterwards she awoke suddenly with a violent pain in the left side of her face, darting through the temple, behind the ear, and along the edge of the trapezius muscle of the same side. From that moment until the 8th October, when she first consulted me, the pain never left her, and scarcely abated at all, except during two or three hours every morning. No cause could be assigned for the attack. There was neither redness, swelling, nor heat of the integuments, nor any inflammation of the gums, but the patient was unable to eat solid food, not so much on account of any tenderness of the teeth, as because the act produced a violent paroxysm of shooting and burning pain over the whole side of the head and neck. The affection was thought to be rheumatic, and a variety of anodyne and rubefacient applications were made to the suffering parts, with no other result, however, than to assuage the pain temporarily.

On hearing these facts, and discovering no other sign of inflammation except pain, I examined, by pressure, the several points where the branches of the trigeminus, and other nerves of the side of the head, become superficial. The following points were acutely sensible to very slight pressure; viz.: the *temporal*, the *mental*, the *mastoid*, and the *occipital*. The *infra* and *supra orbital*, and the *cervical* points were tender, but less so than those just enumerated. Pressure upon the *mental*, *temporal*, and *occipital* produced violent burning or darting pains in all the other points. Upon striking each of the teeth lightly with a file, none of them showed any morbid sensibility except the second inferior molar of the left side, and from it, upon the slightest touch, lancinating pains radiated to most of the points already mentioned. A cavity was soon discovered in the crown of this tooth, but I could not ascertain that its central pulp was exposed, notwithstanding a careful exploration with a pointed probe.

The patient was directed to have the tooth extracted immediately, but the dentist to whom she applied for this purpose insisted upon taking out the adjoining *first* molar, which was not in the least carious. The pain continued without abatement, and, on the next day but one, Mr. Townsend removed the affected tooth. The relief was instantaneous, and so considerable that the patient took advantage of the opportunity to have some other dental operations performed.

The extracted tooth has in its crown a large cavity, which does not, however, appear to have any communication with the central hollow of the organ.

Two days afterwards, (Wednesday, October 12th,) there was no pain, either spontaneous or excited, at any of the spots which had so recently been intolerably sensitive, with the exception of the *occipital*, which was still a little tender. A small flying blister was prescribed to be applied over the seat of pain.

On the 16th instant, or six days after the removal of the diseased tooth, not a vestige of pain remained. Owing to its rapid decrease the prescribed blister had not been applied.

This case has been recorded less on account of any intrinsic interest it may possess, than because it offers an exception to a rule laid down in a recent and very valuable treatise on neuralgia. M. Valleix, the author of that treatise, sets forth the conclusions he arrives at, as the results of his own experience not only, but as legitimate inferences from all the authentic records of the disease, to which he has had access. Speaking of "the state of the teeth" as an alleged cause of tri-facial neuralgia, he states that out of twelve cases of this disease, in which the condition of the teeth is recorded, *nine* were of persons having one or more carious molar teeth. But *four* of these had no dental pain; *six* others submitted to the extraction of the diseased teeth, and had their sufferings rather increased than diminished, and another who had never before had neuralgia, was attacked by it immediately upon the removal of a carious canine tooth. Hence M. V. infers that dental caries must be very rarely the cause of neuralgia.

In a subsequent article, M. Valleix treats of the signs by which odontalgia is to be distinguished from neuralgia of the fifth pair. "A person," he says, "suffering from the former can generally indicate very precisely the starting point of his pain, and the anguish is excessive whenever the tooth, and especially its carious portion, is touched; finally, (and this is the most important of all,) we *cannot excite pain by pressing on the several points of emergence of the nerves, which are so acutely sensitive in neuralgia.*"

The case just recited must clearly be regarded as forming an exception to these propositions, since, in the subject of it, pain in the carious tooth and neuralgic pain began at the same moment, and ended together by the extraction of the decayed tooth. That the pain was really neuralgic, is made apparent by its sudden onset, by the absence of inflammation in the affected region, and above all by the morbid sensibility of the branches of the trigeminus, and of the occipital and cervical nerves.

It is not easy to say in what manner the caries of the tooth produced the neuralgia, since the cavity of the tooth was found to have no communication with that produced by the disease; nor was there any exostosis or other organic affection of either fang.

